# The Impact of Gut Microbiome on Maternal Fructose Intake-Induced Developmental Programming of Adult Disease

**DOI:** 10.3390/nu14051031

**Published:** 2022-02-28

**Authors:** Chien-Ning Hsu, Hong-Ren Yu, Julie Y. H. Chan, Kay L. H. Wu, Wei-Chia Lee, You-Lin Tain

**Affiliations:** 1 Department of Pharmacy, Kaohsiung Chang Gung Memorial Hospital, Kaohsiung 833, Taiwan; cnhsu@cgmh.org.tw; 2 School of Pharmacy, Kaohsiung Medical University, Kaohsiung 807, Taiwan; 3 Department of Pediatrics, Kaohsiung Chang Gung Memorial Hospital, Chang Gung University College of Medicine, Kaohsiung 833, Taiwan; yuu2002@cgmh.org.tw; 4 Institute for Translational Research in Biomedicine, Kaohsiung Chang Gung Memorial Hospital, Chang Gung University College of Medicine, Kaohsiung 833, Taiwan; jchan@cgmh.org.tw (J.Y.H.C.); klhwu@cgmh.org.tw (K.L.H.W.); 5 Department of Urology, Kaohsiung Chang Gung Memorial Hospital, Chang Gung University College of Medicine, Kaohsiung 833, Taiwan; dinor666@ms32.hinet.net

**Keywords:** fructose, hypertension, gut microbiota, developmental origins of health and disease (DOHaD), short chain fatty acid, probiotics, prebiotics, metabolic syndrome

## Abstract

Excessive or insufficient maternal nutrition can influence fetal development and the susceptibility of offspring to adult disease. As eating a fructose-rich diet is becoming more common, the effects of maternal fructose intake on offspring health is of increasing relevance. The gut is required to process fructose, and a high-fructose diet can alter the gut microbiome, resulting in gut dysbiosis and metabolic disorders. Current evidence from animal models has revealed that maternal fructose consumption causes various components of metabolic syndrome in adult offspring, while little is known about how gut microbiome is implicated in fructose-induced developmental programming and the consequential risks for developing chronic disease in offspring. This review will first summarize the current evidence supporting the link between fructose and developmental programming of adult diseases. This will be followed by presenting how gut microbiota links to common mechanisms underlying fructose-induced developmental programming. We also provide an overview of the reprogramming effects of gut microbiota-targeted therapy on fructose-induced developmental programming and how this approach may prevent adult-onset disease. Using gut microbiota-targeted therapy to prevent maternal fructose diet-induced developmental programming, we have the potential to mitigate the global burden of fructose-related disorders.

## 1. Introduction

The developmental origins of health and disease (DOHaD) hypothesis supports that in utero adverse events prime the risk for developing chronic disease later in life [1]. Maternal nutrition plays a crucial role in fetal growth and development. Inappropriate intakes of certain nutrients have been linked to many adult diseases of developmental origins [2]. The consumption of fructose has risen in the last half-century and is thought to play a potential role in the epidemic of hypertension, obesity, diabetes, kidney disease, etc. [3]. Emerging evidence shows increased fructose intake in pregnancy can cause fetal programming and result in a variety of adverse outcomes in offspring, such as hypertension, obesity, diabetes, cardiovascular disease, and non-alcoholic fatty liver disease [4,5].

The major organ for fructose metabolism is the liver, while excessive consumption of fructose can be spilled over to the gut microbiota [6]. Intestinal fructose absorption is limited due to selective absorption [6]. Unabsorbed fructose is converted by the gut microbiome into hydrogen, short-chain fatty acids (SCFAs), methane and carbon dioxide [7]. Accordingly, a high-fructose diet can alter gut microbiome, leading to gut dysbiosis and microbial metabolite disorder. A growing body of evidence supports that maternal diet impacts gut microbiota establishment in offspring [8]. Maternal high-fructose diet has been shown to increase risk for developing adult disease, which is associated with alterations of gut microbiota and its metabolites [9,10,11]. However, there remains gaps in our knowledge about whether early life interventions may target gut microbiota, resulting in protection against adult diseases programmed by maternal fructose exposure.

**Figure 1:**
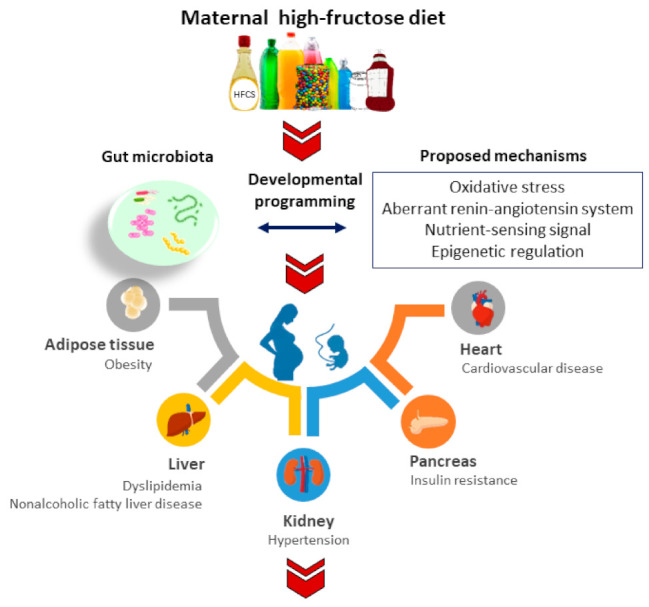
Schematic diagram summarizing the proposed mechanisms linking gut microbiota to maternal fructose-induced developmental programming in different organ systems resulting in various adult diseases in later life.

This review will focus specifically on gut microbiome implicated in the developmental origins of adult disease programmed by maternal fructose exposure. The molecular and mechanistic pathways mediating fetal programming will be a special focus, and their interconnections with gut microbiome will be addressed. Further, the potential of the reprogramming approach targeting gut microbiota to protect progeny against maternal high-fructose diet-induced adult disease will be discussed. A schematic diagram summarizing the adverse impact of a maternal fructose diet on adult offspring and the proposed mechanisms by which gut microbiota may implicate in adult diseases of developmental origins is depicted in Figure 1.

We searched the PubMed/MEDLINE databases for studies published in English between January 1980 to December 2021. Search terms were as follows: “fructose”, “gut microbiota”, “short chain fatty acid”, “blood pressure”, “metabolic syndrome”, “hypertension”, “obesity”, “diabetes”, “fatty liver”, “cardiovascular disease”, “kidney disease”, “developmental programming”, “DOHaD”, “mother”, “maternal”, “pregnancy”, “gestation”, “offspring”, “progeny”, and “prenatal”. Additional studies were then selected and assessed based on appropriate references in eligible papers.

## 2. Maternal Fructose Diet Programs Adult Diseases

Fructose is a structural isomer of glucose and galactose. Although fructose occurs naturally in fruits and vegetables, current fructose consumption is mostly derived from refined sugars and processed foods made up of high-fructose corn syrup (HFCS). Fructose is absorbed in the gut through facilitated glucose transporters 5 (Glut 5) and Glut 2 [6] and almost entirely metabolized in the liver [12]. Today, epidemiological and animal studies have revealed that excessive fructose consumption is a risk factor in the development of obesity and several metabolic disturbances [3]. A systematic meta-analysis recruiting 15 human studies demonstrated that fructose consumption was positively associated with elevated systolic blood pressure (BP), increased fasting blood sugar, and elevated triglycerides [13]. Another meta-analysis study demonstrated sugar-sweetened beverages that contain fructose were associated with a risk of developing hypertension [14].

Notably, both negative and positive effects of fructose consumption have been reported in human studies [15,16,17,18,19]. These controversies arise largely because all sources of fructose are not the same, and not all people respond to fructose in the same way [15,16]. Fructose in fruit tends to be safer because of the additional nutrients and antioxidants in fruit, whereas fructose in high fructose corn syrup (HFCS) and refined sugar is mostly harmful [17,18,19]. Additionally, the response to fructose in young healthy people is much lower than in older obese persons. Therefore, most pooling epidemiological studies that contain various participants as well as fructose from different sources carries great risk for diluting any real findings. Since human studies have not yet established the direct cause-and-effect relationship between excessive fructose consumption and adverse offspring outcomes, it stands to reason that the use of animal models is essential to investigate maternal fructose-induced developmental programming for further translational research.

So far, no information exists regarding the impact of excessive fructose consumption during gestation and/or lactation on offspring health in humans. Given this limitation, much of what is known about the implication of maternal fructose intake on offspring health is only based on experimental animal models.

Recent evidence has emerged from animal models that maternal fructose diet causes various components of metabolic syndrome in their offspring [20,21], including obesity [22], hypertension [22,23,24], dyslipidemia [25], and insulin resistance [22,26]. Additionally, maternal high-fructose consumption is associated with impaired spatial learning and memory [27], and early onset retinopathy [28]. Nevertheless, the role of gut microbiome in the developmental origins of adult disease programmed by maternal fructose exposure remains largely unknown.

## 3. Fructose and Gut Microbiota

Major functions of the gut microbiota include the maintenance of the structural integrity of the gut, host nutrient metabolism, regulation of immune homeostasis, xenobiotic and drug metabolism, and fermentation of non-digestible substrates [29]. It is becoming increasingly obvious that a loss of balance in gut microbiota, termed dysbiosis, is implicated in numerous human diseases. Gut microbiota-derived metabolites are key molecular mediators between the microbiota and the host [30]. Certain metabolites, notably bile acids, SCFAs, branched-chain amino acids, tryptophan derivatives, and trimethylamine N-oxide (TMAO), have been connected to the pathogenesis of metabolic disorders [30]. Emerging data have demonstrated an association between the fructose and gut microbiota dysbiosis in metabolic syndrome-related disorders [31,32]. Although prior research reports that the gut microbiota compositions differ between healthy subjects and patients with metabolic syndrome [33], the causality is still insufficiently demonstrated.

### 3.1. How Fructose Alters Gut Microbiota and Their Metabolites

The tight junctions form a selectively permeable barrier defending the host by avoiding the entry of intestinal microbes and their products. Chronic consumption of fructose is accompanied with a loss of intestinal tight junction proteins, resulting in elevated translocation of endotoxin [34]. A high-fructose diet fed to mice has been shown to result in the development of hyperglycemia, adiposity, dyslipidemia, endotoxemia, and glucose intolerance, which coincided with lost gut microbial diversity in these mice [35]. A high-fructose diet also altered gut microbiota compositions, characterized by a lower abundance of *Bacteroidetes* and a markedly increased proportion of *Proteobacteria*. Another study demonstrated that a high-fructose diet induced steatosis with dyslipidemia and was associated with decreased beneficial microbes *Bifidobacterium* and *Lactobacillus* [36].

Additionally, several lines of evidence indicate fructose is able to mediate microbiota-derived metabolites. First, SCFA levels in plasma from rats fed with a high-fructose diet were reduced [37]. Another line of evidence comes from metabonomic analysis. The metabolic profiling from high-fructose- and salt-fed rats showed the increase of TMAO in urine was associated with metabonomic progression axes, progressing from normal to insulin resistance and hypertension status [38]. This final status of hypertension is an observation regarding mice fed a HFCS-moderate fat diet and displayed anxio-depressive behavior coinciding with altered gut microbiota compositions and tryptophan metabolites [39]. Altogether, these studies indicate that fructose is able to induce gut microbiota dysbiosis in three ways: it disrupts the gut barrier triggering endotoxemia and inflammation; it alters gut microbial profile and diversity; and it influences key microbial metabolites.

### 3.2. The Impact of Maternal Fructose Diet on Gut Microbiome

Much of the work investigating the actions of fructose on gut microbiota has directly studied the fructose-fed animals, yet relatively little data exist on its programming effect on the offspring’s gut microbiota. A summary of animal studies demonstrating the association between gut microbiome, maternal high-fructose intake, and subsequent development of diseases in adult offspring is provided in Table 1 [9,10,40,41,42]. The current review is merely narrowed to a fructose diet starting in the pregnancy and/or lactation period. As shown in Table 1, rats were the dominant animal species being used. The major adverse outcome is hypertension [9,40,41,42].

Adding 10% fructose to the drinking water of pregnant rats significantly altered the maternal microbiome [10]. Notably, there was a significant reduction in *Lactobacillus* and *Bacteroides*; both are commonly known as beneficial microbes. Their female offspring developed adiposity, dyslipidemia, and insulin resistance at 8 weeks of age. These findings were associated with a reduction in the expression of tight junction proteins in the offspring; however, the offspring microbiome was not assessed in this study. Similarly, a maternal high-fructose diet also alters the microbiome in rat offspring. Maternal high-fructose diet-induced hypertension in adult male offspring is related to decreased genus *Akkermansia* abundance [40]. Another study showed adult offspring born to dams that received a 60% fructose diet during pregnancy and lactation displayed an increase in the *Firmicutes* to *Bacteroidetes* ratio [41], a microbial marker of hypertension [42]. A follow-up study identified that maternal plus post-weaning high-fructose diet programs caused hypertension and coincided with a decreased abundance of genera *Lactobacillus, Leuconostoc* and *Turicibacter* [41].

Moreover, a maternal high-fructose diet not only alters microbiota compositions but also their metabolites in adult offspring. An association has been found between maternal high-fructose-induced programmed hypertension and gut microbial metabolites, trimethylamine (TMA), and acetate [40]. TMA is a microbiota-derived precursor of TMAO. Like TMAO, TMA is emerging as a cardiovascular risk marker [43,44]. The major SCFAs produced are acetate, propionate, and butyrate. Evidence shows that SCFAs regulate BP via interacting with SCFA receptors, including G protein-coupled receptor 41 (GPR41), GPR43, and olfactory receptor 78 (Olfr78) [45]. Feeding mother rats with a 60% fructose diet causes elevation of BP in adult offspring, relevant to an increase in plasma acetate level and a decrease in renal GPR41 and GPR43 expression [9]. Considering acetate is a ligand for Olfr78 to induce vasoconstriction and GPR41 exhibits vasodilatory action [46], these findings suggest that SCFAs and their receptors may be involved in maternal high-fructose diet programs leading to hypertension in their offspring.

Abnormalities in early-life gut microbiota were related to a number of adverse offspring outcomes, including obesity [47,48], insulin resistance [47,49], dyslipidemia [49], nonalcoholic fatty liver disease [48], and cardiovascular disease (CVD) [49]. All of these diseases are connected with fructose-induced developmental programming. In the current review, limited information is available about the use of gut microbiota-targeted therapies to study other adult diseases programmed by maternal fructose consumption, such as obesity, liver steatosis, dyslipidemia, insulin resistance, and cardiovascular disease.

Using *Lactobacillus* as a probiotic intervention, previous studies demonstrated it slowed progression of liver steatosis [50] and type II diabetes [51] in fructose-fed rats and mice. Additionally, maternal *Lactiplantibacillus plantarum WJL* treatment prevented adult offspring against CVD [49]. Another study revealed that maternal oligofructose therapy attenuated hepatic steatosis and insulin resistance induced in adult offspring born to dams received high-sucrose/-fat diets [52]. At this point, these studies evaluating the effect of gut microbiota-targeted therapies have only examined the established disease model or developmental models programmed by other insults. There will be a growing need to examine their reprogramming effects in adult diseases related to maternal fructose-induced developmental programming.

Furthermore, prior research demonstrated that excess maternal fructose consumption that caused adverse fetal outcomes was related to increased placental uric acid levels, while treatment of mother mice with the xanthine oxidase inhibitor allopurinol reduced placental uric acid levels and improved fetal weights and serum triglycerides [53]. Given that uric acid is a key mediator in high-fructose intake-related disorders [3] and intestinal microbes like *Lactobacillus* and *Pseudomonas* are known to participate in the metabolism of uric acid [54], probiotics *Lactobacilli* with uric acid-lowering effects targeting the gut microbiota may be a potential therapy for fructose-induced programming in further research.

### 3.3. How Gut Microbiota Links to Common Mechanisms Underlying Fructose-Induced Developmental Programming

In addition to gut microbiota dysbiosis, a number of mechanisms are proposed to be involved in fructose-induced developmental programming and the resulting adult disease. These molecular mechanisms include oxidative stress, aberrant renin–angiotensin system (RAS), nutrient sensing signals, epigenetic regulation, arachidonic acid metabolism pathway, etc. [4,5,14,55,56]. Some aforementioned mechanisms are interrelated to gut microbiota. We will discuss each of these mechanisms in turn.

#### 3.3.1. Oxidative Stress

The fetus is extremely sensitive to oxidative damage during development because of its low antioxidant capacity [57]. A maternal high-fructose diet has been shown to induce various features of metabolic syndrome in adult offspring. Among them, dyslipidemia [58], insulin resistance [26], and hypertension [55,56] have been related to oxidative stress. Oxidative stress can reduce nitric oxide (NO) production by enhancing asymmetric dimethylarginine production (ADMA, an endogenous inhibitor of NO synthase) [59]. Increased plasma ADMA levels and decreased NO bioavailability have been reported in maternal fructose diet-induced programmed hypertension [23].

Conversely, antioxidants can reduce oxidative stress and prevent adult disease of developmental origins [60]. Melatonin is an antioxidant. Its use in pregnancy and lactation has shown to have beneficial effects on hypertension programmed by maternal high-fructose consumption [23]. Because fructose-induced developmental programming induces various features of metabolic syndrome targeting different organs, there is still a lack of reliable data on which organ-specific redox-sensitive signals are responsible for fructose-triggered programming processes.

Fructose-induced developmental programming, apart from oxidative stress, has been linked to gut microbiota. Gut microbial communities have been shown to trigger redox signaling and maintain host–microbiota homeostasis [61]. When an imbalance in the redox state occurs, inflammatory responses may mediate collateral tissue damage and end organ dysfunction [61]. Therefore, oxidative stress seems to work together with gut microbiota behind fructose-induced developmental programming. Attention will be needed to be paid to understanding how gut microbiota interrelates with oxidative stress to trigger organ-dependent programming processes, and whether interventions targeting gut microbiota in pregnancy may also reduce oxidative stress to prevent adult progeny against adult disease of developmental origins.

#### 3.3.2. Aberrant RAS

The RAS is closely connected with adult disease of developmental origins [62]. The RAS is composed of different angiotensin peptides with diverse biological actions mediated by distinct receptors [63]. In general, activation of the classical axis of ACE/angiotensin II (ANG II)/ANG II type 1 receptor (AT1R) triggers vasoconstriction, oxidative stress, and inflammation. Maternal high-fructose diet-induced hypertension is relevant to the aberrant activation of RAS, represented by increases in (pro)renin receptor, angiotensinogen, and angiotensin-converting enzyme (ACE) in the kidneys (minocycline). In contrast, the non-classical RAS, composed mainly by the ACE2/angiotensin-(1-7) (Ang-(1-7))/Mas receptor (MasR)/ANG II type 2 receptor (AT2R), can counterbalance the adverse effects of ANG II. In the high-fructose diet plus 2,3,7,8-tetrachlorodibenzo-p-dioxin (TCDD) exposure model, 3,3-dimethyl-1-butanol (DMB) therapy protected against hypertension coinciding with decreased AT1R and increased AT2R protein abundance [11]. Emerging evidence suggests a bidirectional interaction between the gut microbiome and RAS; gut microbiota-derived metabolites can modulate the gut RAS, while alterations in RAS shape microbiota composition and metabolic activity [64]. Considering maternal fructose consumption altered gut microbiota and the RAS concurrently, more work is required to explore the interaction between gut microbiome and the RAS implicating the pathogenesis of fructose-induced developmental programming.

#### 3.3.3. Nutrient-Sensing Signals

During fetal development, nutrient-sensing signals regulate fetal metabolism in response to maternal nutritional status [65]. Accordingly, disturbed nutrient-sensing signals in pregnancy have a distinctive role in the pathogenesis of adult disease of developmental origins [66]. A number of signals, including silent information regulator transcript (SIRT), AMP-activated protein kinase (AMPK), peroxisome proliferator-activated receptors (PPARs), and PPARγ coactivator-1α (PGC-1α), are related to developmental programming [67]. SIRT1 and AMPK can mediate deacetylation and phosphorylation of PGC-1α, respectively. The downstream signaling effect of PGC-1α is PPARγ, which governs the expression of specific sets of PPAR target genes involved in hypertension of developmental origins [68]. In a maternal and post-weaning high-fructose diet rat model, renal mRNA expression of AMPK, PGC-1α, and PPARs were shown to decrease [41]. On the contrary, resveratrol, an AMPK activator, can mediate these nutrient-sensing signals to activate PPAR target genes and thereby protect offspring against metabolic syndrome-related programmed processes [41]. Additionally, maternal insulin therapy, shown to prevent the elevation of BP in adult offspring born to fructose-fed dams, was associated with enhanced phosphorylated AMPKα2 protein levels [41]. These observations suggest a potential connection between nutrient-sensing signals and gut microbiota underlying fructose-induced developmental programming.

#### 3.3.4. Epigenetic Regulation

The epigenetic modification of genes has emerged as a key mechanism for developmental programming [69]. These modifications include DNA methylation, histone modification, and noncoding RNAs, all of which control gene activation or silencing [70]. Our previous work recognized significant alterations of renal transcriptome in 1-day-old male offspring exposed to maternal high-fructose intake by using next-generation RNA sequencing (NGS) analysis [55]. In total, 2706 differential expressed genes (DEGs) (1214 up- and 1492 down-regulated genes) were identified. Among them, *Cyp2c23, Hpgds, Ptgds* and *Ptges* belonging to arachidonic acid metabolism were involved in maternal high-fructose diet-induced hypertension [56]. Moreover, a number of genes regulating fructose metabolism, fatty acid metabolism, glycolysis/gluconeogenesis, and insulin signaling appear to be regulated by a maternal high-fructose diet in different organs at 1 day of age in our follow-up study [20]. Notably, a maternal high-fructose diet induces differential alterations of gene expression in the brain, kidney, heart, and urinary bladder in progeny. Our NGS results suggest that epigenetic regulation may be involved in the developmental programming of various adult diseases in an organ-specific manner. Additionally, maternal fructose exposure altered the miR-206 expression level in offspring liver that increased promoter methylation at *Lxra* gene [70,71]. Gut microbiota and their metabolites have shown the ability of epigenetic programming of multiple host tissues [72]. The SCFAs can form acetyl-CoA, the substrate for histone acetyltransferase (HAT) enzymes. Further, butyrate is a known histone deacetylase (HDAC) inhibitor. Both scenarios could affect histone modification. Thus, maternal fructose diet-related gut dysbiosis induces epigenetic programming of offspring genes, and corresponding epigenetic mechanisms together with associated gut microbes await further elucidation.

## 4. Reprogramming Strategies Targeted on Gut Microbiota

**Figure 2:**
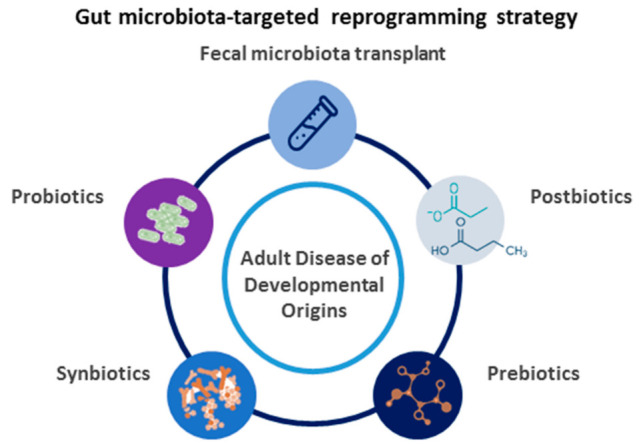
Schematic diagram of the potential gut microbiota-targeted therapies used for adult disease of developmental origins.

Concerning our advanced understanding of developmental programming, it turns out that a therapeutic approach can be shifted from adulthood to early life, even before disease occurs. This is termed as reprogramming [73]. In view of the fact that research into gut microbiota has uncovered a number of interesting contributions to health and disease, researchers are turning their attention to focus on gut microbiota as a potential target for therapeutics [74,75,76]. We propose a diagram to illustrate the potential gut microbiota-targeted therapies as a reprogramming strategy for the prevention of maternal fructose diet-induced adult disease, which is depicted in Figure 2.

### 4.1. Gut Microbiota-Targeted Therapy

The gut microbiome can be targeted with a variety of modalities, including probiotics (i.e., beneficial microbes), prebiotics (i.e., nutrition or food for those beneficial microbes), synbiotics (i.e., mixture of probiotics and prebiotics), postbiotics (i.e., substances produced through the metabolism of the gut microbes), and fecal microbiota transplant.

As reviewed elsewhere [75,76,77,78], gut microbiota-targeted therapy has shown benefits against a broad range of diseases. The widespread clinical use of gut microbiota-targeted treatments are probiotics and prebiotics. Though results from human studies reported beneficial effects of probiotics during pregnancy on maternal outcomes, little is known about the effectiveness in protecting offspring against DOHaD-related disorders. Due to ethical considerations in humans, experiments to target gut microbiota for the prevention of adult disease of developmental origins have provided emerging evidence in animal models. Nevertheless, targeting gut microbiota and derived metabolites remain a flourishing area of research with potential reprogramming interventions for DOHaD-related disorders.

### 4.2. The Use of Gut Microbiota-Targeted Therapy as a Reprogramming Strategy

Here, we summarize studies documenting reprogramming strategies in animal models of fructose-induced developmental programming, focusing on interventions aimed at the gut microbiota. Notably, gut microbiota-targeted therapy will only be restricted to key periods during early development. A review of the literature exposes that currently there are only a few reports on the reprogramming effects of gut microbiota-targeted therapy related to fructose-induced developmental programming. As illustrated in Table 2, rats have dominated in experiments for studying programmed hypertension. Reported reprogramming interventions consist of probiotics, prebiotics, and postbiotics. Though fecal microbiota transplant has also been shown to rescue metabolic syndrome-related phenotypes in a high-fructose diet rat model [79], its reprogramming effect on offspring born to fructose-fed dam has yet to be studied.

Though several probiotic bacteria are associated with health benefits [78,79,80], there is currently no information to suggest their roles on the fructose-induced developmental programming. Only one study documented that *Lactobacillus casei* supplementation during gestation and lactation protects adult male rat offspring against hypertension induced by a maternal high-fructose diet [40]. The beneficial effects of *Lactobacillus casei* supplementation against maternal fructose diet-induced hypertension is related to a decrease in plasma acetate level and renal Olfr78 expression. Considering acetate can induce hypertension by activation of Olf78, SCFA and their receptors are involved in the protective benefits of maternal probiotics treatment. In the same study, long-chain inulin was investigated in regards to their reprogramming effects [40]. Although several types of prebiotics provide various health benefits [76,77], only inulin has been examined in the maternal fructose diet model. Supplementation with inulin during pregnancy and lactation protected maternal high-fructose diet-induced programmed hypertension and was associated with an increased plasma propionate level and renal GPR43 expression [40]. As propionate can activate GPR43 to elicit vasodilatation [45,46], the protective effects of inulin are attributed to gut microbiota-derived metabolite SCFAs. Of note, probiotic and prebiotic therapy show different mechanisms on modulation of gut microbiota despite them exhibiting similar BP-lowering effects [40]. As there are many prebiotic-like components, such as flavonoids, polyphenols, and vitamins, in functional foods, in moving towards their widespread clinical use, there will be a growing need to better understand their reprogramming effects on fructose-induced developmental programming. For example, resveratrol therapy has emerged as a reprogramming agent to protect against several adult diseases of developmental origin [80]. Somewhat surprisingly, there is no research evaluating its protective effects on maternal fructose diet-induced adverse offspring outcomes; such studies are warranted.

The SCFAs have been used as postbiotics as they are fermentation products of polysaccharides by gut microbiota [64]. So far, there is only one report showing that maternal high-fructose diet-induced hypertension can be protected by acetate supplementation [41]. Its protective effects are associated with decreased plasma TMA levels and TMA-to-TMAO ratios, and increased expression of SCFA receptors. As previously mentioned, gut microbiota-dependent TMA and TMAO formation is related to cardiovascular risk. Maternal TMAO administration has been reported to program hypertension in adult male progeny [81]. Conversely, TMA inhibition shows reprogramming effects in fructose-exposed offspring. A structural analogue of choline, 3,3-dimethyl-1-butanol (DMB), showed non-lethal inhibition of TMA and host TMAO formation [82]. Until now, two studies have shown that DMB treatment during pregnancy and lactation protect adult offspring against hypertension programmed by a maternal high-fructose diet [41] or high-fructose diet plus TCDD exposure [42]. The protective effects of DMB therapy are associated with alterations of gut microbes involved in TMA–TMAO metabolism, including the phyla *Firmicutes* and *Proteobacteria*, *Enterobacteriaceae* and *Deferribacteraceae*, and genus *Holdemania* families.

## 5. Conclusions and Future Perspectives

Prior research has indicated that maternal fructose exposure and early life imbalance of gut microbiome might cause adverse offspring outcomes in later life. This review has aimed to highlight the value of gut microbiota-targeted therapy; if applied early, it may protect adult disease of developmental origins programmed by maternal fructose consumption. We fully understand that the presented mechanisms in the current review might not cover the whole picture of the programming effects of fructose related to alterations of gut microbiome. A thorough examination of the relationships between fructose-induced developmental programming and mechanisms behind gut microbiota dysbiosis is worthy of further study. Moreover, what is missing from the literature is a greater understanding of whether maternal or offspring gut microbiota affects developmental programming, due to the inability to longitudinally analyze gut microbiome dynamics at different developmental stages from all reported studies. More attention needs to be paid to analyze how gut microbiome interacts with fructose at the early developmental stage to help explore the causal relationship and elucidate the underlying programmed processes.

Studies to date have documented some evidence regarding how early life gut microbiota-targeted therapies reprogram unexpected fructose-induced programming processes and protect disease in later life; however, almost all studies were focused on hypertension. Given that fructose can program many organs, thus resulting in different phenotypes in adult offspring, there is a pressing need for more studies in their organ-specific reprogramming effects; it is also necessary to evaluate other gut microbiota-related interventions.

Regardless of recent advances in developing potential reprogramming strategies targeting gut microbiota for adult disease of developmental origins, none of them have been translated in human trials. The literature is missing a greater understanding of whether the current uses of probiotics or prebiotic-rich food in pregnant women may also alter gut microbiota and derived metabolites to prevent their children from chronic diseases in adulthood.

In summary, gut microbiota is an important pathophysiological link in early life fructose exposure and developmental programming of adult disease. After a better understanding of fructose-induced developmental programming and this remarkable growth in gut microbiota-target therapy, we expect that translating animal results into optimal clinical practice is a valuable strategy that could reduce the global pandemic of fructose-related disorders.

## Figures and Tables

**Table 1:** Maternal high-fructose diet-induced adult disease of developmental origins related to gut microbiota dysbiosis in animal models.

Animal Models	Species/ Gender	Programming Mechanisms Related to Gut Microbiota	Adverse Offspring Outcomes	References
Maternal 60% fructose diet	SD rat/M	Decreased SCFA receptor GPR41 and GPR43 expression	Hypertension	[9]
Maternal 10% fructose water	Wistar rat/F	Reduced genera *Lactobacillus* and *Bacteroides*	Adiposity, dyslipidemia, and insulin resistance	[10]
Maternal 60% fructose diet	SD rat/M	Reduced genus *Akkermansia* abundance; Increased plasma TMA level	Hypertension	[40]
Maternal plus post-weaning 60% fructose diet	SD rat/M	Decreased abundance of genera *Lactobacillus*, *Leuconostoc*, and *Turicibacter*	Hypertension	[41]
Maternal 60% fructose diet and minocycline administration	SD rat/M	Reduced α-diversity, Decreased genera abundance of *Lactobacillus*, *Ruminococcus*, and *Odoribacter*; Increased abundance of *Akkermansia*; Increased SCFA receptor expression	Hypertension	[42]

SCFA, Short-chain fatty acid. GPR, G protein-coupled receptor. TMA, Trimethylamine.

**Table 2:** Gut microbiota-targeted therapies used as a reprogramming strategy for maternal fructose diet-induced adult disease.

Gut Microbiota-Targeted Therapy	Animal Models	Species/ Gender	Reprogramming Effects	Ref.
*Lactobacillus casei* (2 × 10⁸ CFU/day) by oral gavage from pregnancy through lactation	Maternal 60% fructose diet	SD rat/M	Prevented hypertension	[40]
Long chain inulin (5% *w*/*w*) in drinking water from pregnancy through lactation	Maternal 60% fructose diet	SD rat/M	Prevented hypertension	[40]
Magnesium acetate (200 mmol/L) in drinking water from pregnancy through lactation	Maternal 60% fructose diet	SD rat/M	Prevented hypertension	[41]
DMB (1%, *v*/*v* in drinking water) from pregnancy through lactation	Maternal 60% diet	SD rat/M	Prevented hypertension	[41]
DMB (1%, *v*/*v* in drinking water) from pregnancy through lactation	Maternal 60% fructose diet and TCDD exposure	SD rat/M	Prevented hypertension	[42]

SD, Sprague–Dawley rat; TCDD, 2,3,7,8-tetrachlorodibenzo-p-dioxin; DMB, 3,3-maternal dimethyl-1-butanol.

## Data Availability

Data are contained within the article.
